# Frequent Dental Scaling Is Associated with a Reduced Risk of Periprosthetic Infection following Total Knee Arthroplasty: A Nationwide Population-Based Nested Case-Control Study

**DOI:** 10.1371/journal.pone.0158096

**Published:** 2016-06-23

**Authors:** Ta-Wei Tai, Tzu-Chieh Lin, Chia-Jung Ho, Yea-Huei Kao Yang, Chyun-Yu Yang

**Affiliations:** 1 Department of Orthopaedics, National Cheng Kung University Hospital, College of Medicine, National Cheng Kung University, Tainan, Taiwan; 2 Institute of Biomedical Engineering, National Cheng Kung University, Tainan, Taiwan; 3 Institute of Clinical Pharmacy and Pharmaceutical Sciences, National Cheng Kung University, Tainan, Taiwan; 4 Joy Dental Clinic, Tainan, Taiwan; University of Washington, UNITED STATES

## Abstract

Oral bacteremia has been presumed to be an important risk factor for total knee arthroplasty (TKA) infection. We aimed to investigate whether dental scaling could reduce the risk of TKA infection. A nested case-control study was conducted to compare 1,291 TKA patients who underwent resection arthroplasty for infected TKA and 5,004 matched controls without infection in the TKA cohort of Taiwan’s National Health Insurance Research Database (NHIRD). The frequency of dental scaling was analyzed. Multiple conditional logistic regression was used to assess the frequency of dental scaling and the risk of TKA infection. The percentage of patients who received dental scaling was higher in the control group than in the TKA infection group. The risk for TKA infection was 20% lower for patients who received dental scaling at least once within a 3-year period than for patients who never received dental scaling. Moreover, the risk of TKA infection was reduced by 31% among patients who underwent more frequent dental scaling (5–6 times within 3 years). Frequent and regular dental scaling is associated with a reduced risk of TKA infection.

## Introduction

Total knee arthroplasty (TKA) is currently the best solution for decreasing knee pain and improving function in patients with end-stage osteoarthritis, rheumatoid arthritis, and other rheumatic diseases. As life expectancy has increased, the number of patients requiring TKA is growing exponentially. In 2005, approximately 500,000 TKAs were performed in the United States. This number is expected to increase to 3,500,000 by 2030, according to one projection[1]. Currently, there are more than 20,000 TKAs performed per year in Taiwan. All of the expenses associated with surgery and hospitalization are covered by the National Health Insurance (NHI) system.

Periprosthetic joint infection is the most common complication following TKA, causing patients to suffer from functional loss, additional debridement, and revision surgeries, as well as increased mortality[2]. Delayed or late periprosthetic joint infections are typically caused by hematogenous bacterial spreading. The rate of periprosthetic infection after TKA has been reported to be 0.58–1.56%[3,4] and continues to increase[5]. This situation remains a challenge to the medical care system.

Bacteria present in the oral cavity are the source of 6–13% of TKA infections[6]. These bacteria translocate from the mouth to the bloodstream and cause transient bacteremia. This translocation occurs as a result of not only dental procedures but also daily activities, such as brushing teeth, flossing, and chewing[7]. This “oral bacteremia” occurs more frequently in patients with poor dental condition than in patients with good dental health[8].

Prior recommendations for the prevention of oral bacteremia-related TKA infection focused on prescribing prophylactic antibiotics before dental procedures[9,10]. The current practice guideline released by the American Academy of Orthopaedic Surgeons (AAOS) and the American Dental Association (ADA) in 2012 suggests that advising patients with prosthetic joint implants to maintain appropriate oral condition seems to be more important than prescribing prophylactic antibiotics before any dental procedure[11]. However, the reliable evidence linking poor oral condition to post-TKA infection is still lacking.

Regular dental checkups and tooth scaling to remove plaque and calculus contribute to good oral condition. Good oral health conditions may decrease the risk of bacteremia after tooth brushing, chewing, and scaling[12]. Dental plaque accumulation and gingival inflammation significantly increase the prevalence of bacteremia after daily tooth brushing[8]. Although there is still debate over the effectiveness of regular dental scaling and its optimal frequency in healthy adults[13,14], many dentists provide dental scaling services as part of routine dental care. In Taiwan, the NHI system provides each beneficiary with one dental scaling per six months. Its large-scale database provides good evidence with which to evaluate the effectiveness of dental scaling.

We hypothesized that frequent dental scaling could further decrease the risk of periprosthetic joint infection following TKA. The purpose of this study was to clarify the association between dental scaling frequency and TKA infection risk reduction by employing a nationwide population-based case-control study.

## Materials and Methods

### Data source

Datasets were obtained from Taiwan's National Health Insurance Research Database (NHIRD). Taiwan launched a single-payer NHI program in 1995, and by 2007, 99% of the population was enrolled. The NHIRD contains demographic data on enrollees, information on healthcare professionals and medical facilities, and service records and expenditure claims from inpatient, ambulatory care, and contracted pharmacies for reimbursement purposes[15]. Electronic databases including such data are provided to scientists in Taiwan for research purposes. Data have been anonymized and de-identified prior to analysis. The study protocol was reviewed and approved by the Institutional Review Board of National Cheng Kung University Hospital, Tainan, Taiwan.

### Study population

This study was a nested case-control study. A TKA cohort comprised patients older than 40 years who had undergone TKA from 1999–2002 was identified. Patients were followed from the date they underwent TKA until their death or until 5 years after the incident TKA. Eligible cases were patients who underwent resection arthroplasty for post-TKA infection during the follow-up period; these were defined as cases with inpatient claims with NHI surgery codes (64198B, Removal of prostheses) and concomitant use of systemic antibiotics for at least 7 days. The date on which case patients underwent resection arthroplasty was defined as the index date. Each case was matched with 4 controls from the TKA cohort by age and gender, using the previously proposed incidence density sampling method[16], which has been demonstrated to provide an unbiased estimation of relative risk in a nested case-control study. For controls, the end of 5-year follow-up was set as the index date.

### Cumulative frequency of dental scaling and covariates

Records of the cumulative frequency of dental scaling within 3 years prior to the index date were retrieved from outpatient claims according to NHI procedure codes (91003C, 91004C). In the primary analysis, we compared the risk of infection between patients who had ever and those who had never received dental scaling. We further classified the frequency of dental scaling as 1–4 times and 5–6 times in 3 years in the secondary analysis to determine whether there was a dose-response relationship between dental scaling and infection risk. The Taiwan NHI program provides dental checkups and scaling to all beneficiaries once every six months. Therefore, we defined 5–6 dental scalings in 3 years as a regular dental scaling frequency.

The following covariates were included to assess the study outcomes: age, calendar year, osteoarthritis, rheumatoid arthritis, gout, diabetes mellitus, ischemic heart disease, hypertension, peripheral vascular disease, heart failure, chronic lung disease, hyperlipidemia, ischemic stroke, transient ischemic attack, osteoporosis, and gingival and periodontal disease.

### Statistical analysis

Differences in patient demographic information and other covariates were determined either by the chi-square test or Student’s t-test. Conditional multiple logistic regression was used to calculate the odds ratio (OR) between individuals who received dental scaling and individuals who did not receive dental scaling. All data construction and analyses were performed using SAS 9.3 (SAS Institute, Cary, NC).

## Results

From 1999–2002, a total of 32,391 patients who had recently undergone TKA were included in the TKA cohort. We then identified 1,291 cases that underwent TKA and subsequently required resection arthroplasty because of periprosthetic infection within five years after the primary surgery. A total of 5,004 age- and sex-matched patients were identified as the control group. Regarding past history and comorbidities, the prevalence of rheumatoid arthritis (10.2% in the infection group; 7.4% in the control group), diabetes mellitus (27.3% versus 19.3%), cardiovascular diseases (22.5% versus 19.2%), and chronic lung diseases (22.8% versus 20.1%) were all higher in the infection group than in the control group. There was no significant difference in hypertension, peripheral vascular disease, heart failure, hyperlipidemia, ischemic stroke, transient ischemic attack, or gingival and periodontal diseases between the groups (Table 1).

**Table 1:** Baseline characteristics of patients older than 40 years who had undergone TKA from 1999 to 2002 from the Taiwan National Health Insurance Research Database with and without periprosthetic infection within 5 years.

	Periprosthetic infection* (N = 1,251)	Control (N = 5,004)
**Mean age (SD)**	69.1 (8.0)	69.3 (7.6)
**Calendar year (%)**		
1999	26.1	23.8
2000	26.1	21.2
2001	23.7	22.3
2002	24.1	32.7
**Past medical history (%)**		
Osteoarthritis	87.9	88.6
Rheumatoid arthritis	10.2	7.4
Gout	17.9	15.2
Diabetes mellitus	27.3	19.3
Ischemic heart diseases	22.5	19.2
Hypertension	51.8	50.3
Peripheral vascular disease	1.1	0.8
**Comorbid condition (%)**		
Heart failure	7.0	6.5
Chronic lung disease	22.8	20.1
Hyperlipidemia	15.4	16.1
Ischemic stroke	4.4	4.1
Transient ischemic attack	2.1	1.5
Osteoporosis	21.3	19.4
Gingival and periodontal diseases	17.4	20.2
**Cumulative episodes of dental scaling over a 3-year period (%)** **		
0	73.1	67.8
1–4	19.7	22.4
5–6	7.1	9.9

* NHI surgery codes (64198B, Removal of prostheses) and concomitant use of systemic antibiotics for at least 7 days

** NHI procedure codes (91003C, 91004C)

Overall, the patients in the infection group had undergone less frequent dental scaling (p < 0.0004). In the three-year period before the index date, 73.1% of the patients never visited dental clinics to have dental checkups and scaling (67.8% in the control group). Approximately twenty percent of patients in the infection group had undergone only 1–4 dental scaling procedures in the three years before the index date. Only 7.1% of patients with TKA infection underwent regular dental scaling (10% in the control group).

The results of the multiple conditional logistic regression assessing the risk of TKA periprosthetic infection associated with cumulative episodes of dental scaling, conditional on all baseline characteristics, are shown in Table 2. The primary analysis revealed that patients who had received dental scaling had a 20% lower risk for infection than patients who never received this procedure (adjusted OR, 0.80; 95% confidence interval (CI), 0.68–0.93). We further classified the frequency at which patients received dental scaling into 1–4 times and 5–6 times. Compared with patients who never received dental scaling, patients who had received dental scaling 1–4 times had an OR of 0.84 (95% CI, 0.71–0.99). Moreover, an even lower OR of 0.69 was observed for patients who had received regular dental scaling (95% CI, 0.52–0.89). The results suggested that the more frequently patients underwent dental scaling, the lower their risk of infection.

**Table 2:** Odds of a periprosthetic infection in patients older than 40 years who had undergone TKA from 1999 to 2002 from the Taiwan National Health Insurance Research Database.

Cumulative episodes of dental scaling over a 3-year period (%)	Unadjusted OR (95% CI)	Adjusted OR (95% CI)*
Primary analysis		
No scaling	REF	REF
Received scaling	0.77 (0.66–0.88)	0.80 (0.68–0.93)
Secondary analysis		
No scaling	REF	REF
1–4 times	0.81 (0.69–0.95)	0.84 (0.71–0.99)
5–6 times	0.66 (0.52–0.84)	0.69 (0.54–0.89)

REF: Reference

* Adjusted for all baseline characteristics in Table 1.

## Discussion

The most important finding of this population-based nested case-control study was that frequent dental checkups and tooth scaling may reduce the risk of TKA infection. The risk was reduced by 31% in patients who underwent regular dental scaling compared with patients who received no dental scaling. The possible mechanism of risk reduction is that regular dental checkups and scaling can improve oral health and decrease the risk of transient bacteremia caused by oral flora[17].

Routine dental check-ups help patients identify dental problems and resolve them in the early stage. Dental scaling is a process designed to eliminate the etiologic plaque and calculus to prevent gingival inflammation and further periodontal diseases. It is well-documented that poor dentition increases the risk of bacteremia. A meta-analysis showed this evidence by measuring the gingival index (GI, a scale of 0–3, with 0 indicating normal gingiva and 3 indicating severe inflammation, ulceration, and spontaneous bleeding)[8]. The authors revealed that the patients with gingival disease (GI > 1.5) had a higher risk of bacteremia after tooth brushing (OR: 2.77; 95% CI, 1.5–5.1) than those with relatively healthy gingiva (GI < 1.5). The risk of periprosthetic joint infection after bacteremia was estimated to be 0.1%[18]. However, in the presence of virulent bloodstream infections such as *Staphylococcus aureus*, the risk of periprosthetic joint infection may dramatically increase to as high as 34.1–38.7%[18–21].

Oral bacteria are responsible for approximately 6–13% of cases of TKA infection[6]. Although the magnitude of oral bacteremia required to seed prostheses is unknown, the evidence suggests that maintaining good oral health is important for preventing periprosthetic infection[11]. Our study further explored the beneficial effects of frequent dental checkups and scaling on reducing risk for TKA infection. Although dental scaling causes transient bacteremia[22], there is no evidence that this procedure itself is correlated with periprosthetic joint infection.

A confounding factor regarding this issue is socioeconomic and insurance status. In the United States, although dental caries are common[23,24], individuals with an income below the poverty line are 50% less likely than other individuals to visit a dentist[25]. Lack of dental insurance is also a significant issue in the United States; in particular, the Medicare insurance system does not cover routine dental services such as dental scaling, fillings, and tooth extractions[26], and the Medicaid system currently provides comprehensive dental care in less than half of states[27]. The Taiwan NHI program enrolls nearly all (>99%) inhabitants of Taiwan. It provides dental checkups and scaling to all beneficiaries once per six months. Because basic dental services are fully covered, economic status may have less impact on the ability of individuals to receive dental scaling in Taiwan than in the United States.

Although we adjusted the results extensively with multivariate logistic regression models, there were several limitations and unmeasured confounders in our study. First, the incidence of TKA infection may have been underestimated because we used a strict definition of TKA infection (patients requiring resection arthroplasty due to severe periprosthetic infection plus use of antibiotics). Patients with superficial infection or deep infection treated with only antibiotics and debridement were not captured in our study. Nevertheless, using strict criteria as the outcome may reduce the potential for misclassification bias and provide more valid risk estimation.

Second, there may be some unmeasured confounders associated with the use of the NHIRD. Various types of information regarding patients’ lifestyles and behaviors that could potentially modify infection risk, such as body mass index, dentate status, nutrition, trauma history and cigarette smoking characteristics[28], were not available in our claims database. Furthermore, no information was available regarding self-paid dental services such as dentures and implants. However, current evidence has not confirmed any dental procedure to be a risk factor for periprosthetic joint infection[29].

Third, we also could not control for health awareness. Research has demonstrated that measures of health awareness impact health outcomes ranging from car accidents to melanoma[30]. However, we found no suitable indicator of health awareness. Our results suggested that frequency of dental scaling might be a potential indicator of health awareness; additional studies are needed to validate this possibility.

Last, we could not retrieve information on the causal pathogens of TKA infection from our database, and we could not further investigate the protective effects of dental scaling against specific pathogens. However, these are common limitations of large-scale case-control studies.

Despite our study’s limitations, it also had many strengths. First, our study was the first nationwide study to assess the effect of dental scaling on risk reduction of TKA infection. Second, the database we used (NHIRD) comprised more than 99% of the Taiwanese population. Thus, the TKA cohort in our study has good generalizability. Third, we reported our findings with an extended follow-up period (maximum of 5 years). Finally, we included potential confounders in our database, thereby minimizing potential bias from these factors.

## Conclusion

We found that infrequent dental scaling was associated with periprosthetic joint infection following TKA in Taiwan. This phenomenon is likely attributable to a lack of health awareness. The question of whether regular dental scaling may further reduce the risk of TKA infection requires further investigation.

## References

[1] Kurtz S, Ong K, Lau E, Mowat F, Halpern M. Projections of primary and revision hip and knee arthroplasty in the United States from 2005 to 2030. J Bone Joint Surg Am. 2007 4;89(4):780–5. 17403800 10.2106/JBJS.F.00222

[2] Zmistowski B, Karam JA, Durinka JB, Casper DS, Parvizi J. Periprosthetic joint infection increases the risk of one-year mortality. J Bone Joint Surg Am. 2013 12 18;95(24):2177–84. 10.2106/JBJS.L.00789 24352771

[3] Kurtz SM, Ong KL, Lau E, Bozic KJ, Berry D, Parvizi J. Prosthetic joint infection risk after TKA in the Medicare population. Clin Orthop Relat Res. 2010 1;468(1):52–6. 10.1007/s11999-009-1013-5 19669386 PMC2795807

[4] Suleiman LI, Ortega G, Ong'uti SK, Gonzalez DO, Tran DD, Onyike A, et al Does BMI affect perioperative complications following total knee and hip arthroplasty? J Surg Res. 2012 5 1;174(1):7–11. 10.1016/j.jss.2011.05.057 21816426

[5] Kurtz SM, Lau E, Watson H, Schmier JK, Parvizi J. Economic burden of periprosthetic joint infection in the United States. J Arthroplasty. 2012 9;27(8 Suppl):61–5 e1. 10.1016/j.arth.2012.02.022 22554729

[6] Zimmerli W, Trampuz A, Ochsner PE. Prosthetic-joint infections. N Engl J Med. 2004 10 14;351(16):1645–54. 15483283 10.1056/NEJMra040181

[7] Maharaj B, Coovadia Y, Vayej AC. An investigation of the frequency of bacteraemia following dental extraction, tooth brushing and chewing. Cardiovasc J Afr. 2012 7;23(6):340–4. 10.5830/CVJA-2012-016 22836157 PMC3734757

[8] Tomas I, Diz P, Tobias A, Scully C, Donos N. Periodontal health status and bacteraemia from daily oral activities: systematic review/meta-analysis. J Clin Periodontol. 2012 3;39(3):213–28. 10.1111/j.1600-051X.2011.01784.x 22092606

[9] American Dental A, American Academy of Orthopedic S. Antibiotic prophylaxis for dental patients with total joint replacements. J Am Dent Assoc. 2003 7;134(7):895–9. 12892448 10.14219/jada.archive.2003.0289

[10] Napenas JJ, Lockhart PB, Epstein JB. Comment on the 2009 American Academy of Orthopaedic Surgeons' information statement on antibiotic prophylaxis for bacteremia in patients with joint replacements. J Can Dent Assoc. 2009 7;75(6):447–9. 19627652

[11] American Academy of Orthopaedic Surgeons. Prevention of orthopaedic implant infection in patients undergoing dental procedures: clinical practice guideline. 2012. Available: http://www.aaos.org/Research/guidelines/PUDP/PUDP_guideline.pdf. Accessed June 2016.

[12] Forner L, Larsen T, Kilian M, Holmstrup P. Incidence of bacteremia after chewing, tooth brushing and scaling in individuals with periodontal inflammation. J Clin Periodontol. 2006 6;33(6):401–7. 16677328 10.1111/j.1600-051X.2006.00924.x

[13] Worthington HV, Clarkson JE, Bryan G, Beirne PV. Routine scale and polish for periodontal health in adults. Cochrane Database Syst Rev. 2013;11:CD004625 10.1002/14651858.CD004625.pub4 24197669

[14] Beirne P, Worthington HV, Clarkson JE. Routine scale and polish for periodontal health in adults. Cochrane Database Syst Rev. 2007 (4):CD004625 17943824 10.1002/14651858.CD004625.pub3

[15] NHIRD. National Health Insurance Research Database, Taiwan. Available: http://www.nhri.org.tw/nhird/en/index.htm.

[16] Richardson DB. An incidence density sampling program for nested case-control analyses. Occup Environ Med. 2004 12;61(12):e59 15550597 10.1136/oem.2004.014472 PMC1740694

[17] Lockhart PB, Brennan MT, Thornhill M, Michalowicz BS, Noll J, Bahrani-Mougeot FK, et al Poor oral hygiene as a risk factor for infective endocarditis-related bacteremia. J Am Dent Assoc. 2009 10;140(10):1238–44. 19797553 10.14219/jada.archive.2009.0046 PMC2770162

[18] Uckay I, Lubbeke A, Emonet S, Tovmirzaeva L, Stern R, Ferry T, et al Low incidence of haematogenous seeding to total hip and knee prostheses in patients with remote infections. J Infect. 2009 11;59(5):337–45. 10.1016/j.jinf.2009.08.015 19732794

[19] Murdoch DR, Roberts SA, Fowler VG Jr, Shah MA, Taylor SL, Morris AJ, et al Infection of orthopedic prostheses after Staphylococcus aureus bacteremia. Clin Infect Dis. 2001 2 15;32(4):647–9. 11181131 10.1086/318704

[20] Sendi P, Banderet F, Graber P, Zimmerli W. Periprosthetic joint infection following Staphylococcus aureus bacteremia. J Infect. 2011 7;63(1):17–22. 10.1016/j.jinf.2011.05.005 21663971

[21] Lalani T, Chu VH, Grussemeyer CA, Reed SD, Bolognesi MP, Friedman JY, et al Clinical outcomes and costs among patients with Staphylococcus aureus bacteremia and orthopedic device infections. Scand J Infect Dis. 2008;40(11–12):973–7. 10.1080/00365540802245146 18615359

[22] Waghmare AS, Vhanmane PB, Savitha B, Chawla RL, Bagde HS. Bacteremia following scaling and root planing: A clinico-microbiological study. J Indian Soc Periodontol. 2013 11;17(6):725–30. 10.4103/0972-124X.124480 24554880 PMC3917200

[23] Beltran-Aguilar ED, Barker LK, Canto MT, Dye BA, Gooch BF, Griffin SO, et al Surveillance for dental caries, dental sealants, tooth retention, edentulism, and enamel fluorosis—United States, 1988–1994 and 1999–2002. MMWR Surveill Summ. 2005 8 26;54(3):1–43. 16121123

[24] Dye BA, Fisher MA, Yellowitz JA, Fryar CD, Vargas CM. Receipt of dental care, dental status and workforce in U.S. nursing homes: 1997 National Nursing Home Survey. Spec Care Dentist. 2007 Sep-Oct;27(5):177–86. 17990476 10.1111/j.1754-4505.2007.tb00343.x

[25] Oral health in America: A report of the Surgeon General—executive summary. 2000. Available: http://www.nidcr.nih.gov/DataStatistics/SurgeonGeneral/Report/ExecutiveSummary.htm Accessed June 2016.

[26] Centers for Medicare and Medicaid Services. Dental services. 2012. Available: http://www.medicare.gov/coverage/dental-services.html Accessed June 2016.

[27] Centers for Medicare and Medicaid Services. Dental care. 2012. Available: http://www.medicaid.gov/Medicaid-CHIP-Program-Information/By-Topics/Benefits/Dental-Care.html Accessed June 2016.

[28] Duchman KR, Gao Y, Pugely AJ, Martin CT, Noiseux NO, Callaghan JJ. The effect of smoking on short-term complications following total hip and knee arthroplasty. J Bone Joint Surg Am. 2015;97(13):1049–58. 10.2106/JBJS.N.01016 26135071

[29] Berbari EF, Osmon DR, Carr A, Hanssen AD, Baddour LM, Greene D, et al Dental procedures as risk factors for prosthetic hip or knee infection: a hospital-based prospective case-control study. Clin Infect Dis. 2010 1 1;50(1):8–16. 10.1086/648676 19951109

[30] Dormuth CR, Patrick AR, Shrank WH, Wright JM, Glynn RJ, Sutherland J, et al Statin adherence and risk of accidents: a cautionary tale. Circulation. 2009 4;119(15):2051–7. 10.1161/CIRCULATIONAHA.108.824151 19349320 PMC2744446

